# A Case Study of Zoonotic *Chlamydia abortus* Infection: Diagnostic Challenges From Clinical and Microbiological Perspectives

**DOI:** 10.1093/ofid/ofac524

**Published:** 2022-10-12

**Authors:** Anne-Valérie Burgener, Helena M B Seth-Smith, Sina Kern-Baumann, Ana Durovic, Anette Blaich, Thomas Menter, Elisabeth Bruder, Tim Roloff, Aurélien Martinez, Nicole Borel, Sarah Albini, Irene Hösli, Adrian Egli, Maja Weisser, Vladimira Hinić

**Affiliations:** Division of Infectious Diseases and Hospital Epidemiology, University Hospital Basel, Basel, Switzerland; Division of Clinical Bacteriology and Mycology, University Hospital Basel, Basel, Switzerland; Applied Microbiology Research, Department of Biomedicine, University of Basel, Basel, Switzerland; Department of Obstetrics, University Hospital Basel, Basel, Switzerland; Division of Infectious Diseases and Hospital Epidemiology, University Hospital Basel, Basel, Switzerland; Division of Clinical Bacteriology and Mycology, University Hospital Basel, Basel, Switzerland; Institute of Medical Genetics and Pathology, University Hospital Basel, Basel, Switzerland; Institute of Medical Genetics and Pathology, University Hospital Basel, Basel, Switzerland; Division of Clinical Bacteriology and Mycology, University Hospital Basel, Basel, Switzerland; Applied Microbiology Research, Department of Biomedicine, University of Basel, Basel, Switzerland; Division of Infectious Diseases and Hospital Epidemiology, University Hospital Basel, Basel, Switzerland; Vetsuisse Faculty, Institute of Veterinary Pathology, University of Zurich, Zurich, Switzerland; Vetsuisse Faculty, National Reference Centre for Poultry and Rabbit Diseases, Institute for Food Safety and Hygiene, University of Zurich, Zurich, Switzerland; Department of Obstetrics, University Hospital Basel, Basel, Switzerland; Division of Clinical Bacteriology and Mycology, University Hospital Basel, Basel, Switzerland; Applied Microbiology Research, Department of Biomedicine, University of Basel, Basel, Switzerland; Division of Infectious Diseases and Hospital Epidemiology, University Hospital Basel, Basel, Switzerland; Division of Clinical Bacteriology and Mycology, University Hospital Basel, Basel, Switzerland

**Keywords:** abortion, *Chlamydia abortus*, pregnancy, sequencing, zoonosis, doxycycline

## Abstract

*Chlamydia abortus* is the most common causative agent of abortion in small ruminants, but it is poorly recognized as a human pathogen. In most published case studies, diagnosis remained difficult and often resulted in delayed initiation of therapy. In this case study of severe *C abortus* infection in a pregnant farmer from Switzerland, we highlight the clinical and microbiological diagnostic challenges and provide evidence of a zoonotic epidemiological link.

A previously healthy 33-year-old pregnant woman in her second pregnancy was transferred to our intensive care unit (ICU) with septic shock and severe thrombocytopenia. She was at 19 weeks of gestation and resides on a farm with 200 sheep. Two days earlier, she presented to a peripheral hospital with fever, right-sided flank pain, and polyarthralgia. She was treated with ceftriaxone on clinical suspicion of pyelonephritis. Within 2 days, the patient developed sepsis and was transferred to our tertiary academic center for further therapy. On examination, she was febrile (temperature of 38.6°C), hypotonic (blood pressure 95/53 mm Hg), and tachycardic (heart rate 101 beats per minute). The clinical examination was remarkable for a bilateral conjunctivitis without skin rash. On admission to the ICU, the following laboratory results were abnormal: white blood cell count of 3.42 × 10^9^/L (reference range, 3.5–10 × 10^9^/L), thrombocytes of 18 × 10^9^/L (reference range, 140–450 × 10^9^/L), C-reactive protein of 187 mg/L (reference, <10.0 mg/L), aspartate aminotransferase of 62 U/L (reference 11–34 U/L), and total bilirubin of 20 µmol/L (reference, <15 µmol/L). A thoracic and abdominal computed tomography showed hepatosplenomegaly, but was otherwise normal including the gravid uterus. Ultrasound examination confirmed a viable fetus.

Antibiotic therapy was switched from ceftriaxone to meropenem and trimethoprim/sulfamethoxazole to cover possible infection with *Listeria monocytogenes* and *Coxiella burnetii* and additionally ciprofloxacin to cover for *Francisella tularensis*. The patient’s condition continued to deteriorate with an acute respiratory distress syndrome, and she required invasive ventilation.

Two days after admission, intrauterine fetal death was diagnosed at 19 weeks+1 day of pregnancy and a medical abortion was performed. The histological examination of the placenta showed severe acute basal deciduitis with intervillous abscess formation and septic infarction (Figure 1). No autopsy of the fetus was performed. Swabs from the placenta showed no bacterial growth and broad-range bacterial polymerase chain reaction (PCR) [1] was positive for *Staphylococcus epidermidis*, which was considered a contaminant. Results from routine microbiology (blood and urine cultures and a nasopharyngeal swab for respiratory viruses) were negative. Serological tests for herpes simplex virus, *Brucella* species, *C. burnetii*, *Toxoplasma gondii*, hepatitis A, B, and C viruses, human immunodeficiency virus, *Treponema pallidum*, *Leptospira interrogans*, measles virus, rubella virus, Epstein-Barr virus, and cytomegalovirus showed no active infection. At the time of admission, serological testing for *Chlamydia psittaci* by microimmunofluorescence assay (Anti-Chlamydia-MIF, EUROIMMUN) was negative for immunoglobulin M (IgM) and immunoglobulin G (IgG). Four days after the intrauterine fetal death (and 12 days after initial symptoms), IgG for *C. psittaci* became positive, whereas IgM was not interpretable due to nonspecific fluorescence (Figure 2, Supplementary Table 1). MIF assay for *Chlamydia trachomatis* and *Chlamydia pneumoniae* (located at the same biochip) remained negative at both time points.

**Figure 1.**
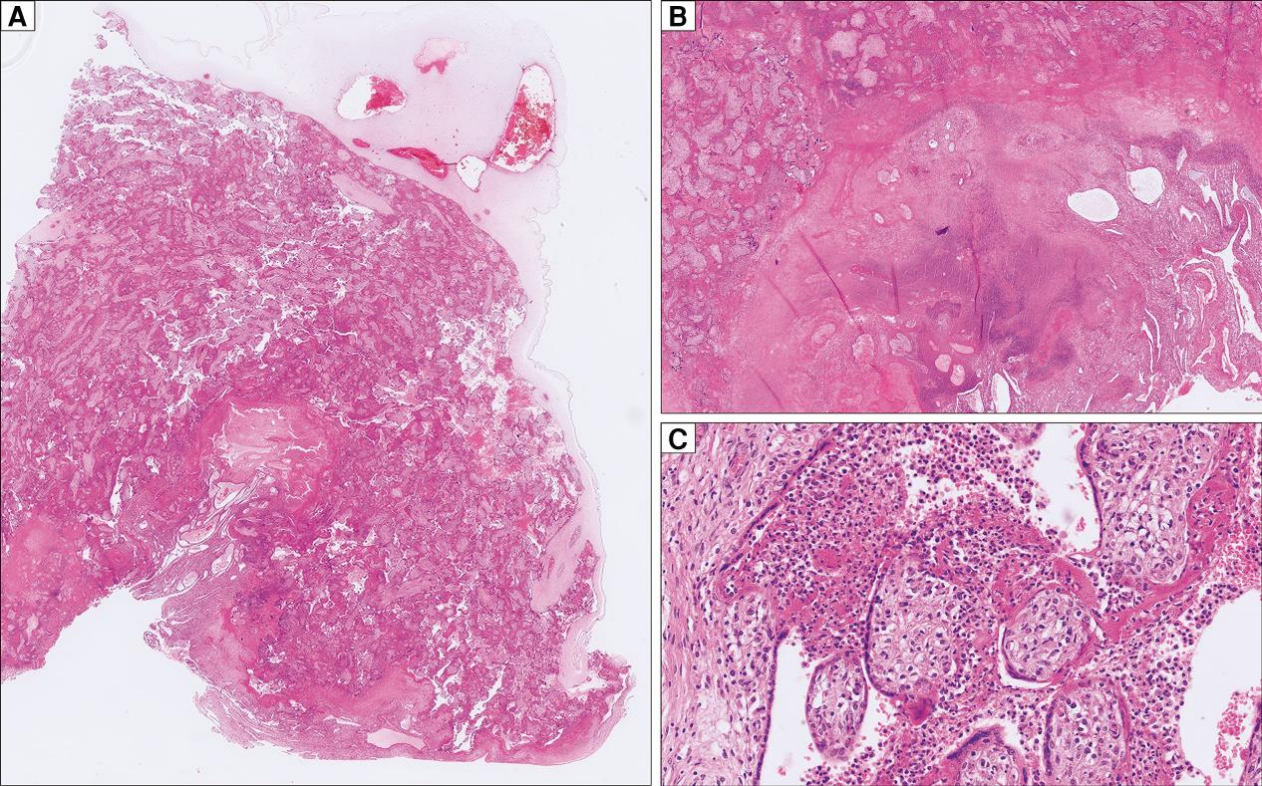
Histological findings of the placenta. *A*, Overview of a basally accentuated inflammatory infiltrate with perivillous fibrin deposits (hematoxylin and eosin [H&E] staining, ×20). *B*, Higher resolution of the basal placenta showing abscessing inflammation of the decidua and infarction of the adjacent placental parenchyma (H&E staining, ×100). *C*, Higher resolution of the placental parenchyma showing florid intervillositis with necrosis of the syncytiotrophoblastic layer of the villi and perivillous fibrin deposition (H&E staining, ×400).

**Figure 2.**
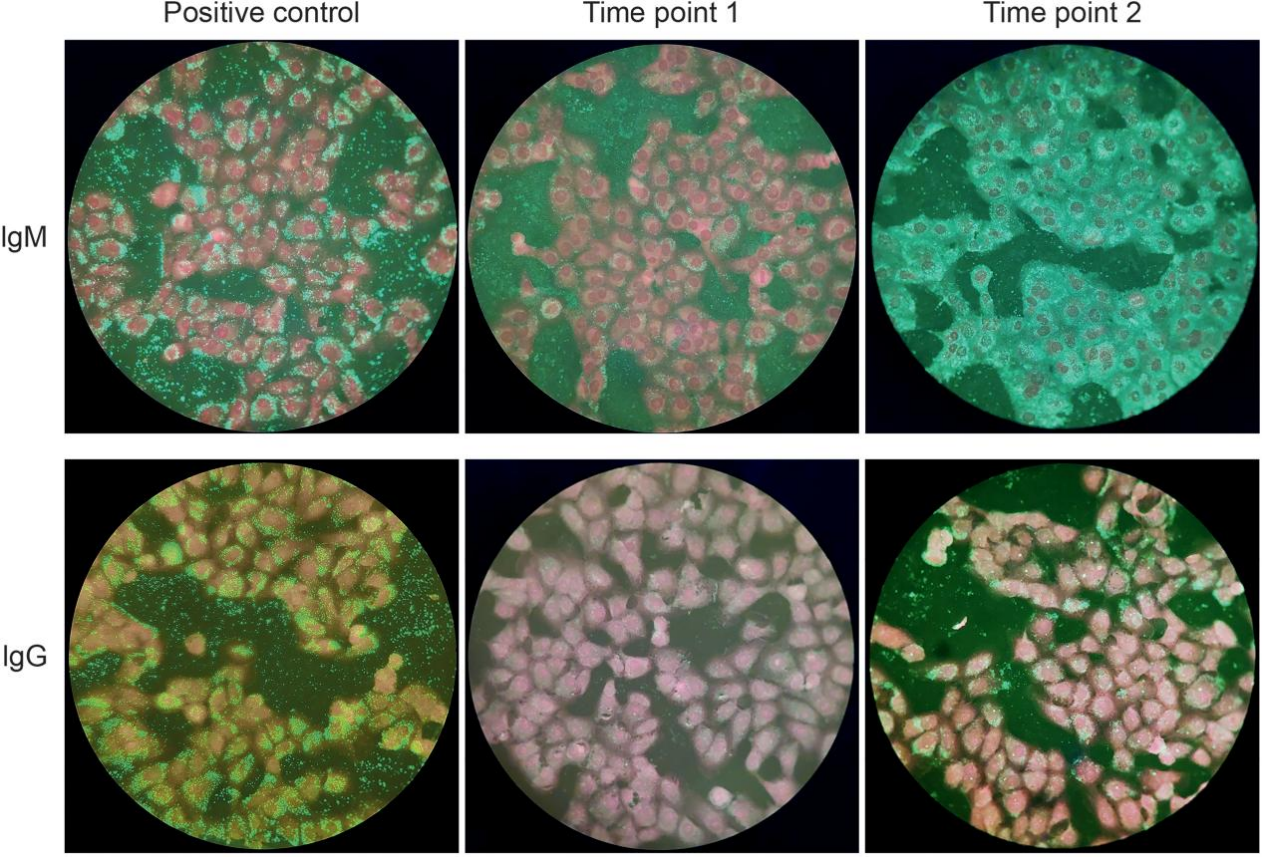
Cross-reactivity with *Chlamydia psittaci* by microimmunofluorescence assay. Time point 1: test performed upon hospital admission; immunoglobulin M (IgM) and immunoglobulin G (IgG) negative. Time point 2: 4 days after intrauterine fetal death (and 12 days after initial symptoms); IgM not interpretable due to presence of unspecific fluorescence, IgG positive. Positive specific fluorescence: presence of granular fluorescence in the cytoplasm and between the cells (eg, positive control). Unspecific fluorescence: differentiation between unspecific and specific fluorescence by comparison of fluorescence of infected cells to uninfected cells (nonspecific binding control).

Cross-reactivity between different *Chlamydia* species in the MIF test has been reported [2]. Based on the positive serology of *C. psittaci* and the suggestive history, we suspected an infection with *Chlamydia abortus*.

Evidence of *C. abortus* was confirmed using a *C. abortus*–specific PCR [3] from DNA extracted by DNeasy Blood and Tissue QIAcube Kit (Qiagen) from both placental swabs. Multilocus sequence typing (MLST) primers specific for *C. abortus* were designed to complement the *Chlamydiales* MLST scheme [4] and were used to analyze the DNA extracted for PCR (Supplementary Table 2). MLST confirmed the diagnosis of *C. abortus* and classified the strain as sequence type (ST) 19 [5]. Metagenomic sequencing of the sample to obtain genomic data from the pathogen retrospectively was attempted from extracted formalin-fixed, paraffin-embedded placental material, as fresh material had been discarded. Using Illumina (NextSeq500) after Illumina DNA prep library creation, only 85635 PE reads resulted from 4 runs. Of these reads, 71 012 were given as unclassified by kraken2 (v 2.0.8-beta) analysis, 14 382 were categorized as human, and of the 12 reads assigned as bacterial, 6 were of *Enterobacterales* origin, 5 chlamydial (4 specifically *C. abortus*), and 1 from *Staphylococcus* spp. By mapping in CLC Genomics Workbench v20.0.2, only 202 read pairs and 27 unpaired reads matched the reference genome (CP018296), and these data were not sufficient to usefully analyze. Oxford Nanopore Technologies (GridIon) was also attempted using an R9.4 flowcells with adaptive sampling with *C. abortus* CP018296 as the reference genome to enrich for, which also provided too few reads to analyze.

When *C. abortus* infection was first suspected, that is, 5 days after admission to the ICU, antimicrobial therapy was switched to oral doxycycline, which led to a rapid clinical recovery of the patient (Figure 3).

**Figure 3.**
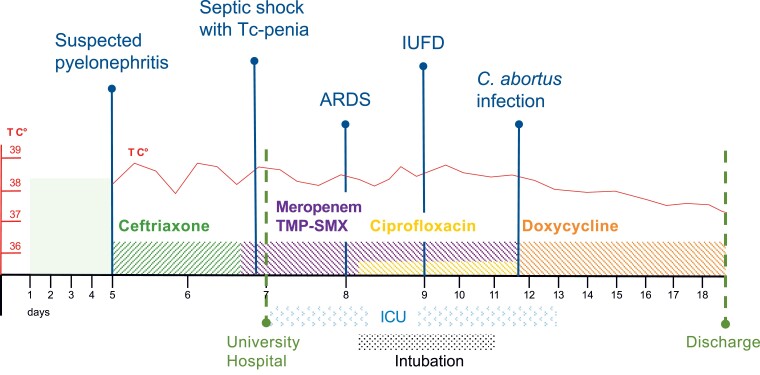
Schematic representation of the clinical course. Diagnoses and antibiotic therapies are indicated. Doses of antibiotic therapies: ceftriaxone, 1 × 2 g/d intravenous (IV), meropenem 3 × 2 g/d IV, trimethoprim-sulfonamide 2 × 240/1200 mg/d oral, ciprofloxacin 2 × 500 mg/d IV, doxycycline 2 × 100 mg/d oral. Red line represents course of temperature in degrees Celsius. Black line represents days. Abbreviations: ARDS, acute respiratory distress syndrome; ICU, intensive care unit; IUFD, intrauterine fetal death; T, temperature; Tc-penia, thrombocytopenia; TMP-SMX,trimethoprim/sulfamethoxazole.

Upon questioning, the patient reported that about 6 days before onset of symptoms, she had helped with the extraction of a dead ovine fetus on her farm. Thus, the cantonal veterinary authority was notified, because ruminant abortions due to *C. abortus* are a notifiable animal disease in Switzerland. A serologic screening of the flock confirmed that 6 of 23 randomly selected animals from that specific farm were serologically positive for *C. abortus*, including the index sheep.

## DISCUSSION

*Chlamydia abortus* is an obligate intracellular bacterial pathogen, formerly known as *C. psittaci* serovar 1. To date, there are no specific serological tests for *C. abortus* approved for diagnosis in humans. Cross-reactivity between different *Chlamydia* species in the MIF test [2] most probably explains the reactive *C. psittaci* IgG in our patient. Infection with *C. psittaci* was not likely in the present case, because the patient did not show signs of an atypical pneumonia, she did not have contact with birds [6, 7], and *C. psittaci* is not commonly associated with abortions in humans [8]. It is possible that some older case reports on “gestational psittacosis” following contact with small ruminants [9, 10] were actually cases of *C. abortus* before this pathogen had been recognized as a new species closely related to *C. psittaci* [11]. As the patient was treated with immunoglobulins for thrombocytopenia, transfusion of *C. psittaci*–specific IgG was theoretically possible.

As a second diagnostic step, we used molecular tests for direct detection of the pathogen.

However, most broad-range bacterial PCRs, including the one we used [1], fail to detect bacteria from the order *Chlamydiales*, because the 16S rDNA sequences of *Chlamydiales* can be distinct from those of other orders, depending on which regions are amplified [12, 13]. Clinicians should be aware of this potential diagnostic gap. Placental swabs from our patient were negative with broad-range bacterial PCR and the diagnosis could only be established by direct detection through a *C. abortus*–specific PCR. This diagnostic workup is usually available in veterinary diagnostic laboratories. That the strain belongs to ST19 suggests a relation to strains from previously characterized European clades [5].

According to study by Borel and colleagues, 18% of all sheep flocks in Switzerland are seropositive for *C. abortus* [14]. Infection control measures in livestock such as testing, sanitary measures, and vaccination are important to prevent infections in humans. As our patient had assisted in the delivery of an aborted sheep fetus on her farm a few days prior to hospitalization, it is likely that the infection with *C. abortus* was caused by direct contact with ovine abortive material or by inhalation of infectious aerosols during lambing. However, several cases of indirect transmission of *C. abortus* have been described in the literature, for example, infection associated with handling contaminated objects such as surfaces, clothing, and footwear [15, 16]. Thus, pregnant women should avoid contact with small ruminants, especially during lambing season.

There are no treatment guidelines for *C. abortus* infections in humans. Antibiotic treatment in the present case was guided by the treatment recommendations for *C. pneumoniae* and *C. psittaci* and included doxycycline. Other therapeutic options described in the literature involve macrolides such as clarithromycin [17]. Historically, tetracyclines have been proven to be teratogenic and are associated with dental and bone abnormalities, neural tube defects, and other organ abnormalities [18, 19]. Doxycycline is a newer tetracycline and has inherited this “tetracycline class effect.” It was formerly classified as a category D by the US Food and Drug Administration and considered contraindicated by many clinicians during pregnancy and in children [20]. However, doxycycline has a lower potential to chelate calcium and is less likely than other drugs in this class to cause permanent tooth discoloration [18, 21]. In addition, exposure to doxycycline in utero could only lead to permanent discoloration of deciduous teeth—after they exfoliate, the condition would completely resolve [22]. Compared to other tetracyclines, doxycycline is administered in lower doses and usually with a shorter treatment duration. Although data are scarce and randomized clinical trials and large pharmacologic studies are not yet available, cumulative evidence has not demonstrated substantial teratogenic side effects with doxycycline treatment [23–25]. Doxycycline is often the most effective treatment for a variety of tropical diseases, zoonotic infections, and contact with biohazardous agents as well as for the empiric treatment of undifferentiated febrile illnesses. The Centers for Disease Control and Prevention recommends doxycycline as first-line therapy for the treatment of Rocky Mountain spotted fever and also anthrax infections, including in pregnant patients and in children, given the potentially fatal outcome [20, 26].

Reports from the United Kingdom indicate that infections with *C. abortus* are responsible for approximately 45% of all diagnosed cases of ovine fetopathy [27]. The actual risk of symptomatic transmission from animals to humans is not known. However, previously published case reports indicate that such infections are associated with high morbidity and mortality [17, 28, 29] . The life-threatening nature to both mother and child therefore outweighs the risks and impact of short-term doxycycline treatment [22].

Consequently, clinicians should initiate antibiotic treatment with doxycycline early in pregnant women with clinical suspicion and a history of contact with ruminants, even if it is not certain that placental infection and fetal death can be prevented after the first clinical signs appear [15, 16].

In conclusion, *C. abortus* is a zoonotic pathogen that causes severe infections during pregnancy and can be life-threatening to the mother and the fetus. Suspicion of the *C. abortus* infection can be raised based on clinical history and laboratory features highlighted in this report. Confirmation of diagnosis relies on specific PCR-based testing. This case reinforces the importance of raising clinicians’ awareness of this pathogen.

## Supplementary Material

ofac524_Supplementary_Data

## Supplementary Data

Supplementary materials are available at *Open Forum Infectious Diseases* online. Consisting of data provided by the authors to benefit the reader, the posted materials are not copyedited and are the sole responsibility of the authors, so questions or comments should be addressed to the corresponding author.

## Notes

***Author contributions.*** A.-V. B. and V. H. wrote the manuscript. H. M. B. S.-S. and T. R. provided multilocus sequence typing data. E. B. and T. M. provided histopathological examination. S. K.-B., A. D., A. B., and A. M. provided additional clinical and diagnostic data. N. B. and S. A. performed the analysis in the veterinary laboratory. M. W., A. E., and I. H. contributed methodological input and critical review of the manuscript.

***Acknowledgments*.** We thank Saša Maksimović for technical assistance and photos of the microimmunofluorescence assay.

***Patient consent.*** The patient consented to publication. A corresponding informed consent is available.
