# Supplementary material for: A Case Study of Zoonotic Chlamydia abortus Infection: Diagnostic Challenges From Clinical and Microbiological Perspectives
Source: Open Forum Infect Dis. 2022 Oct 12;9(10):ofac524. doi: 10.1093/ofid/ofac524 (PMC9605698; doi:10.1093/ofid/ofac524)
Supplement: ofac524_Supplementary_Data [file ofac524_supplementary_data.docx]

| ***C. psittaci* serology** | **Time point 1** | **Time point 2** | **Reference value (titer)** | |
| --- | --- | --- | --- | --- |
| IgM | < 1:10 | not interpretable | < 1:10 = negative | >1:10 = positive |
| IgG | < 1:100 | > 1:100 | < 1:100 = negative | >1:100 = positive |

**Table S1**. **Crossreactivity with *Chlamydia psittaci* serology.** **Time point 1**: test performed upon hospital admission; IgM and IgG negative. **Time point 2**: four days after the IUFD (and 12 days after initial symptoms); IgM not interpretable due to presence of unspecific fluorescence, IgG positive.

**Table S2**. *C. abortus*-specific primers designed for this study, to complement the Chlamydiales MLST scheme (Pannekoek 2008). *Non specific bands still present when amplifying from FFPE placental material.

| **Target** | **F primer name** | **F primer sequence** | **R primer name** | **R primer sequence** | **Expected Size** | **Optimum Tm** |
| --- | --- | --- | --- | --- | --- | --- |
| enoA | Ca_enoAf | cctatgatgaatctcattaatgg | Ca_enoAr | cccaaccgtcaaaatcttcttcag | 444 | 60 |
| fumC | Ca_fumCf | gggctcctgaggtgatgcc | Ca_fumCr | cgcaaatagtgaatcatcttatc | 648 | 67* |
| gatA | Ca_gatAr | ccaccggtatccgaacctaacgc | Ca_gatAf | gccttagagttaagaaatgccg | 508 | 65 |
| gidA | Ca_gidAf | gcttattagagagctctccaggc | Ca_gidAr | agcattttctaatccaaga | 673 | 60* |
| hemN | Ca_hemNf | ggatccatttcggaggagga | Ca_hemNr | cccgaaagaatcttctgatgg | 744 | 60 |
| hflX | Ca_hflXf | gagatttttgctaaccgagct | Ca_hflXr | gtaaaacatcctcatgtaacgc | 531 | 62 |
| oppA | Ca_oppAf | atgcgcaagatatcaatggg | Ca_oppAr | ggcaaagtttggtgtaactcgc | 586 | 65 |
